# Guiding Principles for Surgical Pathways: A Tool for Improving Outcomes and Patient Safety

**DOI:** 10.3389/fpubh.2022.869607

**Published:** 2022-04-08

**Authors:** Matteo Bolcato, Daniele Rodriguez, Anna Aprile

**Affiliations:** Legal Medicine, University of Padua, Padua, Italy

**Keywords:** safety, Patient Blood Management (PBM), lean management, surgery, organizational

## Abstract

Surgical activity is an important aspect for the management of health and safety processes and from an organizational perspective is one of the most complex activities performed in hospitals. It is often a defining and high value feature for any healthcare facility while being one of the most high-risk procedures for patients with the highest number of avoidable adverse events. To ensure effective management of surgical pathways, they need to be considered from the perspective of clinical governance which takes a global approach to planning and management with the goal of improving safety and quality for patients. This paper contains the main features of this objective outlined within the document issued subsequent to the State-Regional Italian Government conference. This regulatory effort includes effective recommendations to make surgical pathways safer and more efficient with particular reference to lean management, patient blood management and patient safety.

## Introduction

Millions of surgical procedures are carried out daily worldwide. Surgical activity is of central importance in terms of managing health and safety processes (1) and is one of the most organizationally complex activities performed in hospitals. It is often a defining and high value feature for any healthcare facility accounting for the largest cost center while being one of the most high-risk activities for patients, creating the highest number of avoidable adverse events (2).

Efficient surgical pathway management needs to be considered from the perspective of clinical governance which takes a global approach to organizing healthcare services through planning, the objective of which must be to improve safety and quality for patients (3).

**Figure 1:**
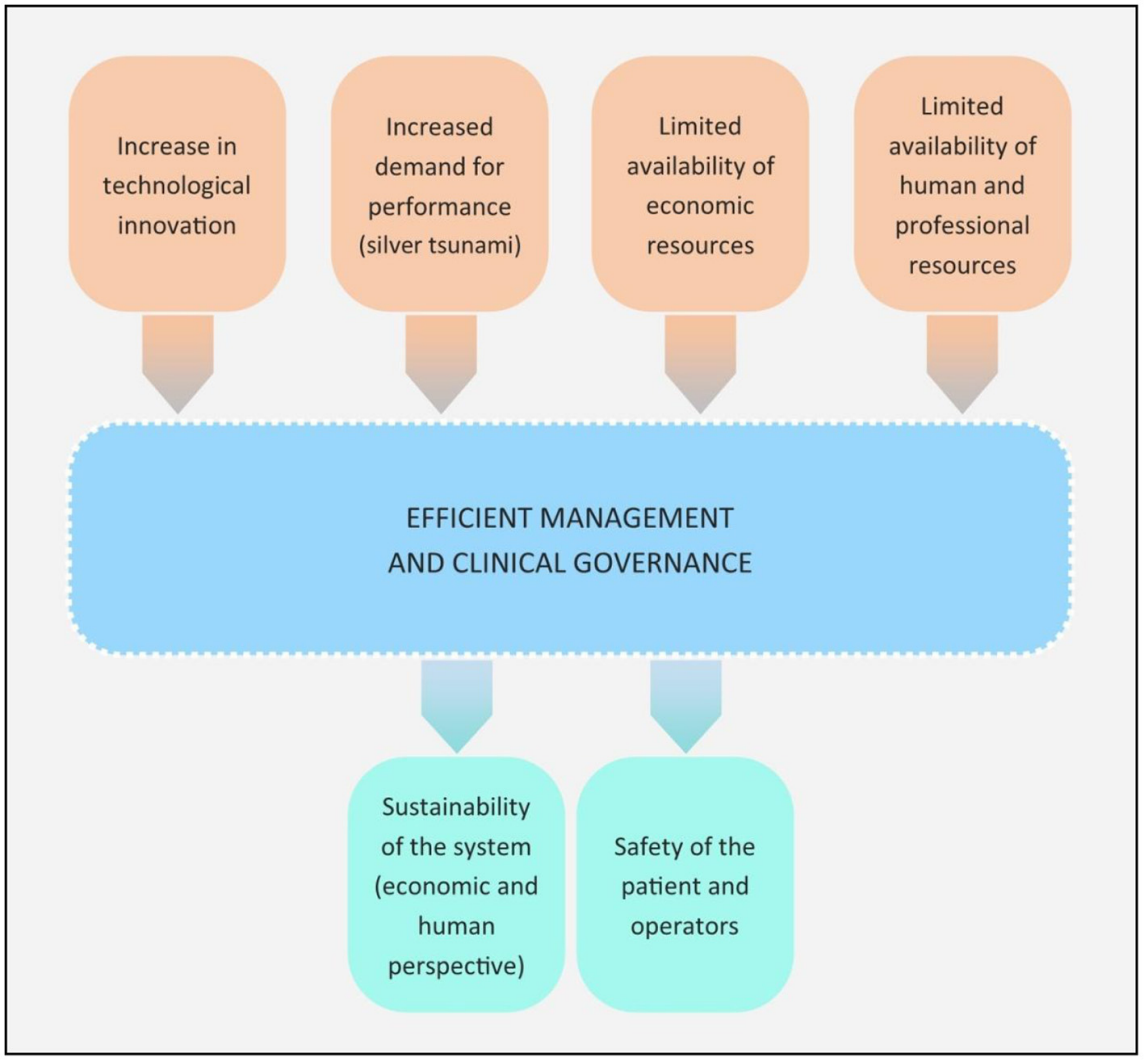
Innovative factors within the current healthcare context.

In recent decades, Western countries have noted a continual growth in demand for surgical treatment. This phenomenon, known as the “silver tsunami”, has been associated with an increase in the average age of the population, an increase in the availability of technological innovations for use in surgical pathways and an imbalance between financial and human resources with respect to the increased service requirements of a rapidly changing population (4) (Figure 1). These factors, innovative in nature and intensity, require management by governance processes.

## Materials and Methods

The present study aims to present the main innovations produced by the document issued in Italy called “Guidelines for the management of the planned surgical patient path” better specified below. A mini-review of the literature available through the use of the PubMed database was also implemented. The words “surgery” and “safety” were searched, and the “full text” and “English” filters were applied. No time limit has been set. The last search was done on December 10, 2021. PubMed's “similar articles” section and the references of the selected articles were used to broaden the search. The screening included a first phase of analysis of the titles and abstracts as a second phase of revision of the complete texts by the three authors in order to isolate articles that could add elements of interest to the suggestions indicated in the recommendations document.

### Guiding Principles for Governing the Planned Surgical Patient Pathway

The Italian Constitution makes provision for the protection of health and these legislative powers are shared between the State and the 20 regions in which the nation is administratively divided. The State determines essential levels of care (LEA) which must be guaranteed nationwide. Each region independently plans and manages public healthcare within its territory. The “Standing Conference on Relations between the State and Regions” is a collective body coordinating activities of shared interest.

It is worth noting that this Standing Conference has drawn up specific planned surgical pathway guiding principles nationwide. In the past, the Ministry of Health and the Prime Minister had issued regulations primarily of a general nature governing LEA and waiting lists.

The document approved by the State-Regional Conference on 9th July 2020 is entitled “Guiding principles for governing the planned surgical patient pathway” (5) [LSP]. The purpose of this document is to define and explain the surgical patient pathway by identifying indispensable features such as activities, roles, timing and responsibilities. Furthermore, risk factors and tools are reported which are useful for defining organizational methods and guarantee the proper functioning of the pathway itself, from the need for surgery to patient discharge, ensuring therefore hospital-territory continuity of care (6).

The main goals outlined in the document are:

- Determining the most suitable management and organizational method to govern the complexity of surgical blocks.
- Defining roles, responsibilities and duties of various professional figures.
- Defining activities that form the pathway.
- Optimizing the use of human, technical, equipment and logistical resources to pursue the goal.
- Aligning operational standards.
- Identifying training strategies.

The LSP describe the planned surgical patient pathway and offer recommendations which are briefly outlined herein.

### Levels of Responsibility and Organization

The LSP map out a chain of practices and activities which start from central government and extend to those involved locally in healthcare authorities. In Italy, healthcare is organized on a regional basis and each region in turn controls local health authorities. The document proposes the creation of a national watchdog which governs the planned surgical patient pathway.

The LSP describe the local organization background and outline the reference framework. Basic requirements for a healthcare authority are a) management needs to guide all stages of the process, b) the creation of professional groups with specific functions, c) the required skills and expertise to achieve the goals must influence the choice of professional figure to include in each group.

Each healthcare authority has a Strategic Group which is responsible for achieving the overall objective of the re-organization, a Planning Team which is assigned to planning and implementing the strategies developed by the aforementioned group, and an Operations Group composed of two practitioners who are responsible for planning operating suites and crisis management.

To establish each group's operational remit, a detailed analysis of the types of surgical treatment required by the target population is essential, thereby determining which pathways to focus on or how to distribute resources.

Below are some key points that the recommendation document contains in order to better organize the operational phase.

### The Peri-Operative Pathway

**Figure 2:**
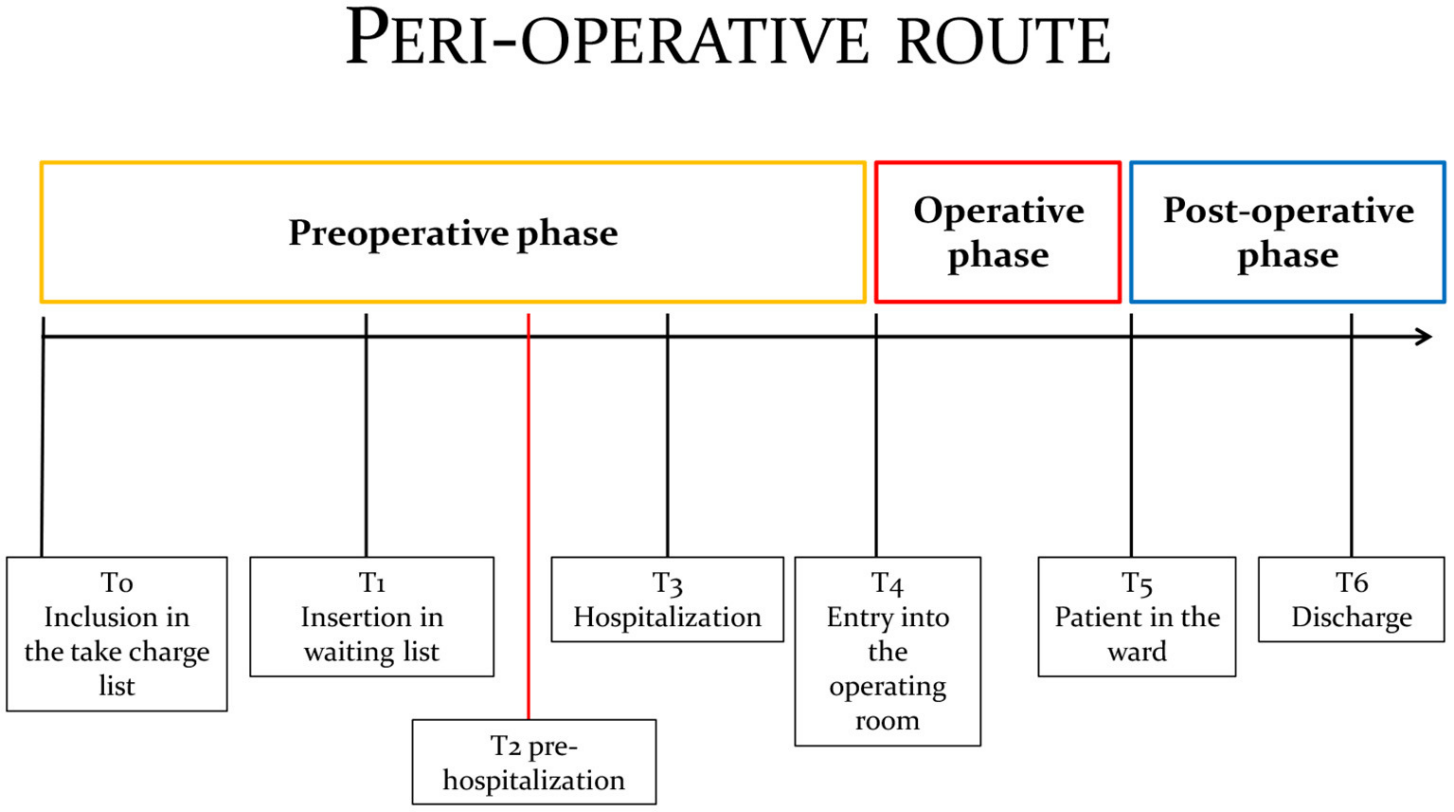
The Perioperative pathway.

The LSP outline the best feasible pathway within a healthcare facility to take charge of a patient (7) as shown in Figure 2. The pathway begins following a specialist's referral for a surgical procedure and concludes when the patient is discharged. The pathway, as shown in the figure, is divided into phases so that the patient can be presented with a plan indicating the progression and timeline of the chosen pathway.

### The Pre-operative Phase

A comprehensive understanding of the number and characteristics of patients awaiting planned surgical hospitalization is a fundamental part of process governance. It is important to regulate the way in which patients are placed on the waiting list by inserting only those ready for surgery, followed therefore by a take charge list. The take charge list comprises patients who have undergone a specialist visit, the results of which require surgery, but who are not in the condition for immediate surgery.

The take charge moment does not always coincide with insertion on the waiting list because there may be a need to administer drug treatment, therapy or wait for a set time for observation or assessment before the operation. Adjusting the surgical activity schedule is important so as to set a definite hospitalization time to perform the operation, as and when the patient is inserted in the waiting list. The proposed hospitalization triggers the patient's eventual Pre-hospitalization activation as well as the Perioperative Risk Optimization and Management Planning Tool [PROMPTs]. This aims to limit unnecessary tests and consultations and evaluate the surgical grading level and the patient's physiological condition (e.g., ASA).

Available resources may now be planned namely organizational, clinical and care-giving. This is necessary in order to perform the operation with the greatest level of efficacy, efficiency, safety and performance quality (8).

An operating room schedule should be compiled each week and submitted to those involved in the process; a schedule could also be drawn up daily (9–11).

The operating room schedule should include the following information:

- The patient's identification data.
- Which operating room will be used.
- Pathology and type of operation foreseen.
- Operation start time and planned duration.
- Operating team.
- The type of anesthesia selected based on the preoperative evaluation.
- The side of the operation.
- The position of the patient.
- The need for hemocomponents.
- Access to Intensive Care or Recovery Room for any potential allergies.
- Hygiene-sanitary rating of the operation.
- Surgical equipment, devices and technology to be used.
- Availability of technicians to perform the operation.

### Pre-hospitalization

Pre-hospitalization consists of patient evaluation prior to hospitalization and has these key objectives:

- Evaluating the patient's general condition and any clinical-organizational measures to initiate during the waiting period.
- Determining perioperative risks.
- Optimizing the patient's condition to reduce perioperative risks.
- Informing the patient about the operation's features and subsequent phases to give them a comprehensive picture of the entire treatment process.
- Outlining the best perioperative management strategy to the patient.
- Reducing hospitalization duration and improving surgical activity scheduling.

In an effort to optimize the pathway, reduce problems and humanize treatment, a personalized approach to taking charge of the patient is preferable when planning the procedure. The Pre-hospitalization pathway is activated to determine surgical risks and confirm that the patient is operable. Furthermore, it enables practitioners to assess the patient's suitability for surgery and, where applicable, further examine them to identify the most suitable treatment setting and provide the patient support in terms of information, choice and the eventual need to administer drugs to improve his or her condition. It is preferable that the patient accesses the healthcare facility as little as possible during the Pre-hospitalization phase. Pre-hospitalization should be carried out with due regard for two key factors: suitability and timing.

### The Post-operative Phase

The postoperative phase embraces activities needed to discharge the patient and involves checking and monitoring the conditions of the patient undergoing surgery and managing adverse events. Every healthcare authority is obliged to identify the responsibilities of healthcare practitioners and provide criteria and management methods for the Post-operative patient; it is essential that the appropriate level of care be decided (12) for each patient.

Human resources and responsibilities for the surgical management pathway will therefore need to be defined, especially with regard to clinical care activities, the expertise of staff employed, the briefing and debriefing communication method between health-care staff in the operating suite and recovery room, available equipment to assess the patient's clinical conditional on arrival and on discharge, methods and the criteria for assistance in the ward and the general specification and risk management criteria.

### Discharge and Take Charge During the First 30 Days: The Territory's Role

The discharge of a hospitalized patient is part of the process which necessitates prompt organization and planning in order to reduce the time a patient spends away from home and maximize the availability of beds to admit new patients.

It is therefore important to prepare the patient to be discharged without delay by:

- Making sure the patient is adequately informed.
- Facilitating access throughout outpatient facilities.
- Promoting interdisciplinary collaboration in the hospital.
- Guaranteeing hospital-territory continuity of care.
- Monitoring outpatient procedures and minor complications.
- Reducing Post-operative hospitalization and eventual rehabilitation.
- Determining average follow-up times for each operation.

## Discussion

Surgical pathway organization and management is of considerable importance from a financial, human and patient safety viewpoint.

To ensure knowledge and understanding of the clinical-care and clinical-surgical process phases, which are alike yet distinctive, all the available tools focusing on prevention, identification, analysis and clinical risk handling for patients and practitioners, such as incident reporting, FMEA-FMECA, root cause analysis etc., should also be applied within the pre- and postoperative phases. The objective is to detect, report and monitor critical points and key moments to ensure governance and safety throughout the peri-operative pathway. This, in turn, will simultaneously ensure efficacy and efficiency by specifically focusing on certain aspects along the process such as:

- Patient Blood Management.
- Safety of electro-medical equipment.
- High risk drugs.
- Management of organizational and technological emergencies.

Adopting risk control models in the process increases the flow of information and facilitates proper planning and scheduling when taking charge of a patient. There is ample evidence of the need to adopt arrangements for reporting adverse events and near misses (13–15). These arrangements would enhance organizational processes as well as setting up multidisciplinary analyses of both reactive (RCA) and proactive events such as SEA and clinical audits. This process, which also takes place by systematic comparisons with known standards, guidelines and best practice, could facilitate the detection of deviations from care reference standards. It would also permit the implementation of timely improvements and allow the impact of the corrective measures introduced to be monitored. The benefits of these measures are multiple:

- Improving practice: creating real benefits in patient care and service provision.
- Developing openness to change.
- Ensuring the quality of care.
- Listening to patients and understanding their response and expectations.
- Developing guidelines and protocols.
- Reducing to a minimum errors or damage to the patient.

In 2009 The Ministry for Health published and in 2013 updated specific documents such as the “Safety Manual for operating rooms: recommendations and checklists”. The LSP stand in continuity with the “Manual” and have repeatedly reiterated its contents incorporating the “recommendations”, the checklist routine and have a common interest in the principles of clinical risk management. The LSP note that it is essential to plan specific clinical risk management models along the pathway, with the aim of avoiding and/or intercepting risk factors. With reference to surgical pathway risk management, it urgently calls for the need to map out all activities the patient undergoes throughout the entire process to detect eventual critical points and shared control methods.

The LSP often refer to the principles and goals of clinical risk management, such as the safety of patients and healthcare professionals, as well as the means for implementing the promotion of a safety ethos, incident reporting, clinical audit, FMEA-FMECA and root cause analysis. The LSP acknowledge that the safety of patients and practitioners is directly proportional to proper organization of activities between the various professional figures who participate in the care process. Consequently, based on clinical evidence (16–18) safety management within the process must be multi-disciplined and multi-professional.

Constant attention is given to a proactive organizational approach and the creation of a healthcare pathway is in fact recommended (19–21). The aim is the continuous improvement of patient care with the purpose of boosting clinical outcomes, hospital performance and safety. As a result, clinical governance practices should be adopted which put the focus on quality and safety within the planning and management of healthcare services and caring for the needs of citizens, whilst at the same time enhancing the role and responsibilities of all healthcare professionals who work in this field.

A key aspect of clinical risk management is the attention given by the LSP to the recognition of Patient Blood Management (PBM). The PBM program is essentially a blood resource plan aimed at ensuring an individual's safety and care (22, 23). PBM is a multi-modal and multi-disciplinary approach that aims to improve clinical results of anemic patients or at risk of anemia (24, 25). Compliance with PBM is considered significant along with three other aspects that could at first appear greater considering their innate multiple risk factors: electro-medical equipment safety, high risk drugs, management of technological and organizational emergencies.

The recognition given by the LSP to PBM follows in the wake of other Italian regulatory sources and other authoritative international deliberations. In Italy, PBM principles are fully adopted in the National Blood Centre guidelines enacted by the Ministry of Health dated 19 January 2017, in line with article 25, clause 5 of the Ministry of Health's decree dated 2 November 2015 (26). Furthermore, the World Health Organization has also reiterated the urgent need to implement PBM worldwide (27).

The LSP often cite “Lean Management” which is “an improvement approach aimed at continual improvement to quality of care processes by means of detecting and eliminating steps that do not add value to the patient such as interruptions, delays and mistakes” (28). This approach has already been implemented within the English healthcare system and is known as “The productive operating Theatre program – TPOT”, where its positive role specifically within the field of surgery is emphasized (29, 30).

## Conclusions

The literature appears to be in agreement with many of the points contained by the LSP as it promises an efficient management of the surgical path in the perspective of clinical governance, combining two systems, clinical risk management and lean management, both aimed at the quality of care provided, with attention to the safety of the assisted person and the optimization of available resources. These objectives are achievable through the adoption of structured organizational paths that include, among others, Pre-hospitalization and the application of Patient Blood Management.

## Author Contributions

MB and DR contributed to conceptualization and contributed to the original draft preparation. AA contributed to methodology, validation, contributed to supervision, and final approval of the manuscript. All authors listed have made a substantial, direct, intellectual contribution to the work, and approved it for publication. All authors contributed to the article and approved the submitted version.

## Conflict of Interest

The authors declare that the research was conducted in the absence of any commercial or financial relationships that could be construed as a potential conflict of interest.

## Publisher's Note

All claims expressed in this article are solely those of the authors and do not necessarily represent those of their affiliated organizations, or those of the publisher, the editors and the reviewers. Any product that may be evaluated in this article, or claim that may be made by its manufacturer, is not guaranteed or endorsed by the publisher.
